# Effect of multidisciplinary team care on survival of oesophageal cancer patients: a retrospective nationwide cohort study

**DOI:** 10.1038/s41598-021-92618-w

**Published:** 2021-06-24

**Authors:** Yuan-Chun Huang, Pei-Tseng Kung, Shang-Yun Ho, Yeu-Sheng Tyan, Li-Ting Chiu, Wen-Chen Tsai

**Affiliations:** 1 Department of Health Services Administration, China Medical University, No. 100, Sec. 1, Jingmao Road, Taichung, 404060 Taiwan; 2 Department of Medical Imaging, Changhua Christian Hospital, Changhua, Taiwan; 3 School of Medical Imaging and Radiological Sciences, School of Medicine, Chung Shan Medical University, Taichung, Taiwan; 4 Department of Medical Imaging, Chung Shan Medical University Hospital, Taichung, Taiwan; 5 Department of Biological Science and Technology, National Chiao Tung University, Hsinchu, Taiwan; 6 Department of Healthcare Administration, Asia University, Taichung, Taiwan; 7 Department of Medical Research, China Medical University Hospital, China Medical University, Taichung, Taiwan

**Keywords:** Health care, Medical research, Oncology, Risk factors

## Abstract

Oesophageal cancer is the sixth leading cause of cancer death worldwide. This nationwide study analyses the survival results of oesophageal cancer under multidisciplinary team (MDT) care. We enrolled oesophageal cancer patients diagnosed between 2010 and 2015 with follow-up for at least 1 year. This study performed propensity score matching with a ratio of 1:1 between MDT participants and non-MDT participants. We performed conditional Cox proportional hazards model to research relative risk of survival and associated factors of survival. The adjusted survival curves were plotted. 8184 newly diagnosed oesophageal cancer patients were included. The favourable survival factors include participant status of MDT, gender, monthly salary, urbanization level, other catastrophic illness, stage of cancer, treatment methods, and service volume of physicians (*P* < 0.05). MDT participants showed lower risk of death (HR = 0.73; 95% CI 0.67–0.79). Further stratification analysis revealed that the incorporation of an MDT reduced the death risk of patients with stages 2, 3, and 4 cancer, with the greatest reduction observed in patients with stage 3 cancer (HR = 0.72; 95% CI 0.67–0.79). The risk of death was lower for oesophageal cancer patients who enrolled in MDT care.

## Introduction

Globally, oesophageal cancer has the ninth highest incidence rate among all cancer types and is the sixth most common cause of cancer death^1^. Despite with ever-improving medical treatment, studies have revealed a low 5-year survival rate among oesophageal cancer patients^2–4^.

After the promulgation of the Cancer Control Act in Taiwan, the Ministry of Health and Welfare, initiated the Complete Cancer Care Quality Improvement Project in 2005 to promote cancer-prevention education, measures, and screening, provide education training on medical personnel, boost the quality of cancer treatment, and assist hospitals in the establishment of a multidisciplinary team (MDT) care model for cancer. An MDT care plan concerns the planning, assessment, review, and analysis of cancer treatment and care; consultation on nutrition, psychological issues, and medicine; health education; rehabilitation; preparation for discharge; continuous care after discharge; and integrative diagnosis and treatment of cancer^5–8^.

Due to its effect in improving clinical care outcomes according to the literature, the implementation of the MDT treatment strategy has been increasing in a number in places including European countries, the United States, and Australia^9^. However, only one study has focused its discussion of MDT care on the effect of improve on the survival rate of oesophageal cancer patients. That study was conducted in one British medical institution from 1991 and 2003; due to the small study population comprising 144 patients and before–after study design, it exhibited various research limitations and insufficient generalisability^10^.

Studies discussing cancers, such as oral, gastric, lung, colorectal, breast, and ovarian cancers, have revealed a conducive effect of MDTs on clinical care outcomes^5,7,11–16^. A study comparing patients with oral cancer who were managed and who were not managed by an MDT showed a lower death risk in those managed by an MDT^11^. A study of gastric cancer revealed the effect of MDTs in improving compliance with treatment guidelines and reducing inappropriate treatment recommendations, which thereby increased the survival of patients with gastric cancer^17^. Research in patients with stage 3 and stage 4 lung cancer showed a significantly lower death risk in those incorporating an MDT into the treatment plan than in those who did not^5^. A study of colorectal cancer also revealed that patients with colorectal cancer who incorporated an MDT into the treatment plan had a lower death risk than did those who did not, and that such a difference was particularly prominent among patients with stage 4 colorectal cancer^18^. According to Liao et al., colorectal patients incorporating an MDT into the treatment had fewer emergency department visits than did those without an MDT, indicating the higher quality of care received by those with an MDT^19^.

However, few studies have also revealed no significant effect of MDT intervention on the survival results. Research on lung and metastatic rectal cancer has shown no effect of MDT intervention on survival results improvement of cancer patients^20,21^. Other studies have noted the difficulties in assessing the effectiveness of the MDT treatment strategy; thus, this topic requires more research efforts^8,9^. Several American scholars suggested that future research should emphasise the various dimensions of the MDT treatment strategy, including its effectiveness and its relationship with patient survival^22^.

Overall, in addition to the appeals from scholars worldwide, the National Comprehensive Cancer Network had listed the MDT treatment strategy as one of the basic treatments for oesophageal cancer^23^. However, at the time of writing the present paper, only one study of MDT intervention focused on the survival of patients with oesophageal cancer^10^. The current medical evidence is insufficient for verifying the effect of MDT intervention on the survival results of oesophageal cancer patients, warranting further research on the topic. Therefore, based on data from the National Health Insurance Research Database (NHIRD) and Taiwan Cancer Registry Database (TCRD), a nationwide retrospective cohort research was conducted to research the effect of MDT intervention in improving the survival rate of oesophageal cancer patients.

## Results

### Features of oesophageal cancer patients adopting and not adopting the MDT treatment strategy

Before matching (Table 1), bivariate analysis revealed significance differences in the socioeconomic factor (monthly salary), environmental factor (urbanization level), health status (comorbidities), service volume of the physician, and hospital level between patients with oesophageal cancer with and without the cancer MDT treatment strategy (*p* < 0.05). According to Table 1, a higher proportion of patients with a high monthly salary decided to incorporate an MDT for oesophageal cancer treatment, with the highest proportion observed among those with a monthly salary between NT$36,301 and NT$45,800 and higher than NT$45,800. A high proportion of patients with oesophageal cancer incorporating an MDT was observed among those who lived in an area with urbanization levels 2, 3, 4, or 7. Regarding the health status (measured based on Deyo’s Charlson Comorbidity Index [CCI] and the presence of other catastrophic illnesses), patients with a favourable health status showed a higher tendency to adopt the MDT treatment strategy. Most of the patients received treatment in medical centres (60.39%). Compared with those visiting medical centres, a higher proportion of patients visiting regional and district hospitals adopted the MDT treatment strategy (medical centre: 32.09%; nonmedical centre: 43.71%). Regarding the service volume of the physician, patients whose physicians had a medium volume of service exhibited the highest tendency to adopt the MDT treatment strategy (39.73%).

**Table 1:** Bivariate and logistic regression analysis: MDT participants and non-participants.

Variables	Total		Non-MDT		MDT		χ^2^	Adjusted			*P* value^b^
	N	%	n_1_	%	n_2_	%	*P* value^a^	OR	95% CI		
**Total**	8184	100.00	5181	63.31	3003	36.69					
**Gender**							0.300				
Female	517	6.32	316	61.12	201	38.88		–	–	–	–
Male	7667	93.68	4865	63.45	2802	36.55		0.88	0.73	1.07	0.193
**Age at diagnosed (year)**							0.015				
< 45	690	8.43	443	64.20	247	35.80		-	-	-	-
45–54	2601	31.78	1601	61.55	1000	38.45		1.12	0.94	1.34	0.219
55–64	2789	34.08	1755	62.93	1034	37.07		1.04	0.87	1.24	0.690
65–74	1253	15.31	803	64.09	450	35.91		1.00	0.82	1.23	0.971
≧ 75	851	10.40	579	68.04	272	31.96		0.86	0.69	1.07	0.185
**Monthly salary (NTD)**							< 0.001				
≦ 17,280	2269	27.72	1495	65.89	774	34.11		–	–	–	–
17,281–22,800	3395	41.48	2158	63.56	1237	36.44		1.10	0.98	1.24	0.096
22,801–28,800	630	7.70	387	61.43	243	38.57		1.15	0.96	1.39	0.139
28,801–36,300	711	8.69	462	64.98	249	35.02		1.03	0.86	1.23	0.767
36,301–45,800	761	9.30	427	56.11	334	43.89		1.50	1.26	1.78	< 0.001
≧ 45,801	418	5.11	252	60.29	166	39.71		1.33	1.07	1.66	0.011
**Urbanization level**							< 0.001				
Level 1	1855	22.67	1109	59.78	746	40.22		1.00	–	–	–
Level 2	2356	28.79	1474	62.56	882	37.44		0.82	0.72	0.93	0.002
Level 3	1524	18.62	1012	66.40	512	33.60		0.72	0.62	0.83	< 0.001
Level 4	1346	16.45	906	67.31	440	32.69		0.68	0.58	0.79	< 0.001
Level 5	238	2.91	125	52.52	113	47.48		1.21	0.91	1.60	0.191
Level 6	447	5.46	282	63.09	165	36.91		0.81	0.65	1.01	0.066
Level 7	418	5.11	273	65.31	145	34.69		0.76	0.61	0.96	0.019
**Other catastrophic illness**							0.023				
No	7782	95.09	4905	63.03	2877	36.97		1.00	–	–	–
Yes	402	4.91	276	68.66	126	31.34		0.86	0.69	1.08	0.199
**Charlson Comorbidity index**							0.001				
0	4734	57.84	2926	61.81	1808	38.19		1.00	–	–	–
1	1924	23.51	1229	63.88	695	36.12		0.92	0.82	1.03	0.151
2	844	10.31	558	66.11	286	33.89		0.86	0.73	1.00	0.056
≧ 3	682	8.33	468	68.62	214	31.38		0.76	0.63	0.91	0.003
**Cancer stage**							< 0.001				
I	343	4.19	222	64.72	121	35.28		1.00	–	–	–
II	1521	18.59	905	59.50	616	40.50		1.18	0.92	1.52	0.188
III	4397	53.73	2775	63.11	1622	36.89		1.01	0.80	1.28	0.926
IV	1923	23.50	1279	66.51	644	33.49		0.86	0.67	1.10	0.235
**Service volume of physicians**							< 0.001				
Low (< 25%)	2030	24.80	1320	65.02	710	34.98		1.00	–	–	–
Middle (25–75%)	4158	50.81	2506	60.27	1652	39.73		1.28	1.15	1.44	< 0.001
High (> 75%)	1996	24.39	1355	67.89	641	32.11		1.04	0.90	1.19	0.636
**Hospital level**							< 0.001				
Medical centers	4942	60.39	3356	67.91	1586	32.09		1.00	–	–	–
Non-medical centers	3242	39.61	1825	56.29	1417	43.71		1.68	1.52	1.86	< 0.001
**Hospital ownership**							0.063				
Non-public	5650	69.04	3539	62.64	2111	37.36		1.00	–	–	–
Public	2534	30.96	1642	64.80	892	35.20		1.02	0.92	1.14	0.655

*MDT* multidisciplinary team, *OR* odds ratio, *NTD* new Taiwan dollar.

### Effect of MDT on the survival of oesophageal cancer patients

Propensity score matching was conducted at a ratio of 1:1. Logistic regression was employed to establish a model; the dependent variable was whether the patient adopted the MDT treatment strategy, and the independent variables comprised demographic factors (sex and age), socioeconomic factor (monthly salary), environmental factor (urbanization level), health status (CCI, other catastrophic illnesses, and oesophageal cancer stage), and characteristics of the main hospital that the patient visited (hospital level and hospital ownership). Logistic regression analysis was conducted to obtain the propensity score of each patient, followed by propensity score matching at a 1:1 ratio. The final research sample included 2953 patients with oesophageal cancer in the experimental group (patients adopting the MDT treatment strategy) and 2,953 patients in the control group (patients not adopting the MDT treatment strategy) (in Table 2).

**Table 2:** Bivariate analysis of factors affecting MDT participant status after propensity score matching.

Variables	1: 1 matching						SMD
	Total		Non-MDT		MDT		
	N	%	n_1_	%	n_2_	%	
**Total**	5906	100.00	2953	50.00	2953	50.00	
**Gender**							
Female	373	6.32	178	6.03	195	6.60	0.071
Male	5533	93.68	2775	93.97	2758	93.40	0.005
**Age at diagnosed (year)**							
< 45	486	8.23	248	8.40	238	8.06	0.042
45–54	1966	33.29	990	33.53	976	33.05	0.061
55–64	2046	34.64	1026	34.74	1020	34.54	0.035
65–74	865	14.65	418	14.16	447	15.14	0.029
≧ 75	543	9.19	271	9.18	272	9.21	0.061
**Monthly salary (NTD)**							
≦ 17,280	1606	27.19	834	28.24	772	26.14	0.034
17,281–2,2800	2421	40.99	1202	40.70	1219	41.28	0.016
22,801–28,800	472	7.99	231	7.82	241	8.16	0.024
28,801–36,300	510	8.64	261	8.84	249	8.43	0.040
36,301–45,800	598	10.13	283	9.58	315	10.67	0.065
≧ 45,801	299	5.06	142	4.81	157	5.32	0.019
**Urbanization level**							
Level 1	1460	24.72	741	25.09	719	24.35	0.047
Level 2	1715	29.04	839	28.41	876	29.66	0.041
Level 3	1020	17.27	511	17.30	509	17.24	0.008
Level 4	872	14.76	434	14.70	438	14.83	0.040
Level 5	204	3.45	102	3.45	102	3.45	0.004
Level 6	342	5.79	177	5.99	165	5.59	0.021
Level 7	293	4.96	149	5.05	144	4.88	0.064
**Charlson Comorbidity index**							
0	3610	61.12	1835	62.14	1775	60.11	0.019
1	1368	23.16	683	23.13	685	23.20	0.018
2	525	8.89	245	8.30	280	9.48	0.027
≧ 3	403	6.82	190	6.43	213	7.21	0.010
**Other catastrophic illness**							
No	5673	96.05	2845	96.34	2828	95.77	0.003
Yes	233	3.95	108	3.66	125	4.23	0.075
**Cancer stage**							
I	236	4.00	116	3.93	120	4.06	0.080
II	1154	19.54	558	18.90	596	20.18	0.049
III	3215	54.44	1618	54.79	1597	54.08	0.029
IV	1301	22.03	661	22.38	640	21.67	0.004
**Hospital level**							
Medical centers	3155	53.42	1571	53.20	1584	53.64	0.050
Non-medical centers	2751	46.58	1382	46.80	1369	46.36	0.026
**Hospital ownership**							
Non-public	4092	69.29	2025	68.57	2067	70.00	0.042
Public	1814	30.71	928	31.43	886	30.00	0.012

*MDT* multidisciplinary team, *NTD* New Taiwan Dollar.

**Figure 1:**
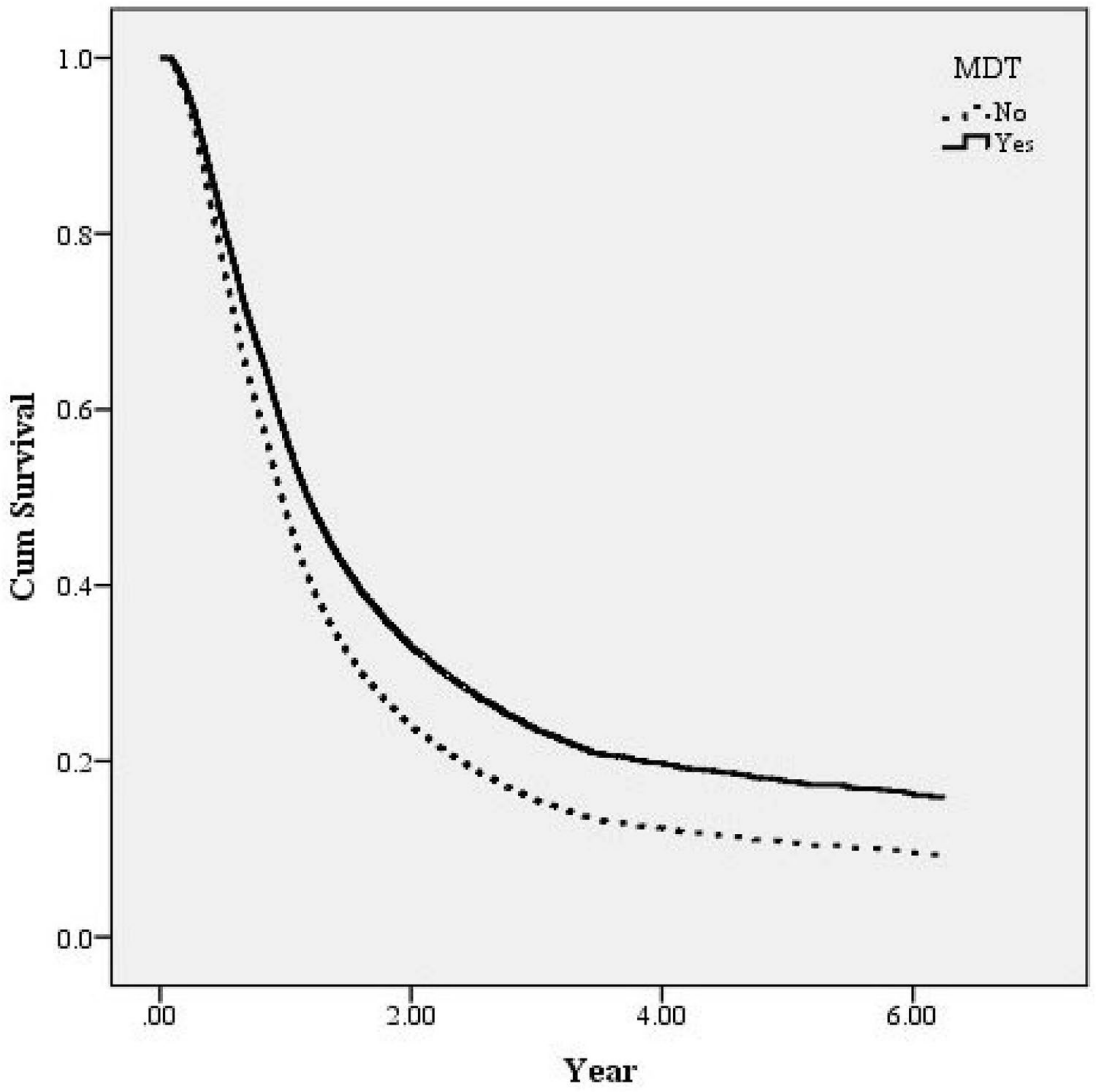
Survival curves of esophageal patients according to MDT participant status. The cumulative survival of esophageal patients among 2953 MDT patients and 2953 non-participants. The survival curves were controlled by gender, age, monthly salary, urbanization level, comorbidities, other catastrophic illness, level of hospital, hospital ownership, service volume of attending physicians. The survival rates of MDT participants were significantly higher than those of MDT non-participants (adjusted HR = 0.73, 95% CI 0.67–0.79).

**Figure 2:**
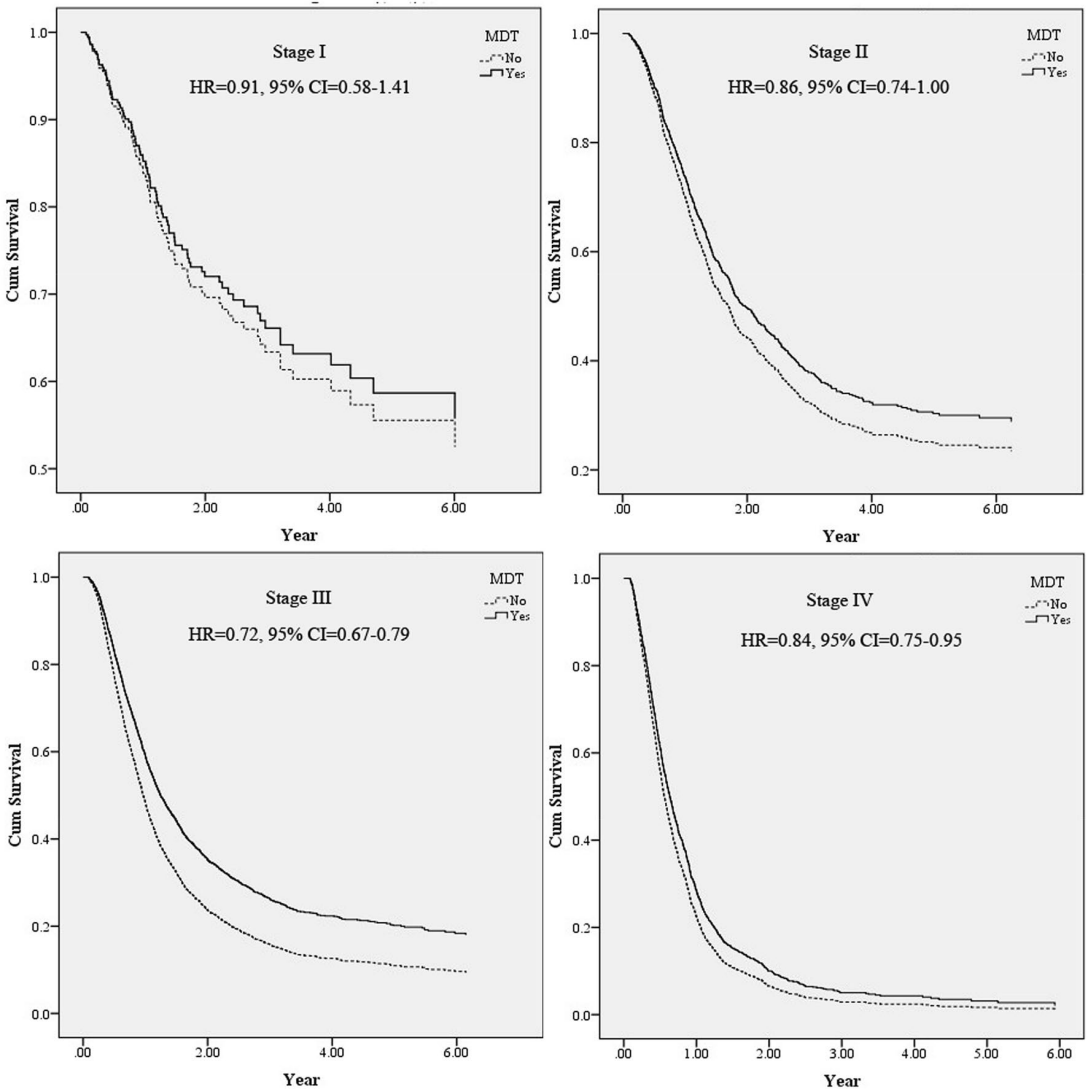
Stratification analysis of the patients according to the oesophageal cancer stage showed the incorporation of an MDT significantly reduced death risk for patients with stages 2, 3, and 4 cancer; the reduction was particularly marked for patients with stage 3 cancer (HR = 0.72; 95% CI 0.67–0.79).

According to Table 3, the death rate was lower in patients adopting the MDT treatment strategy (72.10%) than in those not adopting the MDT treatment strategy (76.36%). A conditional Cox proportional hazard model was employed for statistical analyses to explore the survival rates of those with and without an MDT. As shown in Table 3, the death risk of patients adopting the MDT treatment strategy was 0.73 times that of patients not adopting the MDT treatment strategy (95% confidence interval [CI] 0.67–0.79). With relevant variables controlled for (Fig. 1), adjusted survival curves were generated for patients with and without an MDT. These patients were further divided according to the oesophageal cancer stage for stratification analysis, which showed that the incorporation of an MDT significantly reduced death risk for patients with stages 2, 3, and 4 cancer; the reduction was particularly marked for patients with stage 3 cancer [hazard ratio (HR) = 0.72; 95% CI 0.67–0.79; Fig. 2].

**Table 3:** Analysis of MDT participant status and other factors affecting survival of esophageal patients.

Variables	Total		Survival		Death		*P* value^a^	Adjusted			*P* value^b^
	N	%	n_1_	%	n_2_	%		HR	95% CI		
**Total**	5906	100.00	1522	25.77	4384	74.23					
**MDT participant status**							< 0.001				
No	2953	50.00	698	23.64	2255	76.36					
Yes	2953	50.00	824	27.90	2129	72.10		0.73	0.67	0.79	< 0.001
**Gender**							< 0.001				
Female	373	6.32	145	38.87	228	61.13		–	–	–	–
Male	5533	93.68	1377	24.89	4156	75.11		1.45	1.01	2.09	0.043
**Age at diagnosed (year)**							0.001				
< 45	486	8.23	108	22.22	378	77.78		–	–	–	–
45–54	1966	33.29	464	23.60	1502	76.40		1.29	0.88	1.89	0.189
55–64	2046	34.64	584	28.54	1462	71.46		1.19	0.84	1.68	0.333
65–74	865	14.65	233	26.94	632	73.06		1.30	0.90	1.87	0.163
≧ 75	543	9.19	133	24.49	410	75.51		1.33	0.83	2.15	0.239
**Monthly salary (NTD)**							< 0.001				
≦ 17,280	1606	27.19	316	19.68	1290	80.32		–	–	–	–
17,281–22,800	2421	40.99	625	25.82	1796	74.18		0.74	0.55	1.01	0.047
22,801–28,800	472	7.99	147	31.14	325	68.86		0.76	0.49	1.18	0.214
28,801–36,300	510	8.64	140	27.45	370	72.55		0.65	0.45	0.94	0.023
36,301–45,800	598	10.13	196	32.78	402	67.22		0.60	0.29	1.22	0.159
≧ 45,801	299	5.06	98	32.78	201	67.22		0.42	0.23	0.75	0.003
**Urbanization level**							0.310				
Level 1	1460	24.72	404	27.67	1056	72.33		–	–	–	–
Level 2	1715	29.04	437	25.48	1278	74.52		0.93	0.62	1.41	0.737
Level 3	1020	17.27	283	27.75	737	72.25		1.02	0.56	1.84	0.958
Level 4	872	14.76	195	22.36	677	77.64		0.98	0.51	1.90	0.954
Level 5	204	3.45	47	23.04	157	76.96		0.51	0.29	0.91	0.022
Level 6	342	5.79	81	23.68	261	76.32		0.68	0.40	1.16	0.159
Level 7	293	4.96	75	25.60	218	74.40		1.19	0.64	2.19	0.584
**Other catastrophic illness**							0.591				
No	5673	96.05	1457	25.68	4216	74.32		–	–	–	–
Yes	233	4.96	65	27.90	168	72.10		1.78	1.16	2.74	0.009
**Charlson Comorbidity index**							0.280				
0	3610	61.12	917	25.40	2693	74.60		–	–	–	–
1	1368	23.16	363	26.54	1005	73.46		1.02	0.78	1.33	0.891
2	525	8.89	134	25.52	391	74.48		0.98	0.67	1.43	0.912
≧ 3	403	6.82	108	26.80	295	73.20		0.99	0.58	1.69	0.963
**Cancer stage**							< 0.001				
I	236	4.00	144	61.02	92	38.98		–	–	–	–
II	1154	19.54	445	38.56	709	61.44		1.18	0.70	2.01	0.533
III	3215	54.44	824	25.63	2391	74.37		2.75	1.78	4.26	< 0.001
IV	1301	22.03	109	8.38	1192	91.62		6.49	3.88	10.85	< 0.001
**Treatment**							< 0.001				
Surgery	476	8.06	218	45.80	258	54.20		–	–	–	–
Radiotherapy	303	5.13	33	10.89	270	89.11		2.41	1.66	3.49	< 0.001
Chemotherapy	247	4.18	44	17.81	203	82.19		2.62	1.76	3.89	< 0.001
Surgery + radiotherapy	287	4.86	59	20.56	228	79.44		1.09	0.76	1.56	0.647
Surgery + chemotherapy	283	4.79	58	20.49	225	79.51		1.96	1.36	2.82	< 0.001
Radiotherapy + chemotherapy	1649	27.92	375	22.74	1274	77.26		0.93	0.72	1.20	0.552
Surgery + radiotherapy + chemotherapy	2661	45.06	735	27.62	1926	72.38		0.78	0.60	1.00	0.051
**Service volume of physicians**											
Low (< 25%)	1492	25.26	284	19.03	1208	80.97		–	–	–	–
Middle (25–75%)	3109	52.64	807	25.96	2302	74.04		0.96	0.83	1.11	0.561
High (> 75%)	1305	22.10	431	33.03	874	66.97		0.81	0.67	0.97	0.023
**Hospital level**							< 0.001				
Medical centers	3155	53.42	865	27.42	2290	72.58		–	–	–	–
Non-medical centers	2751	46.58	657	23.88	2094	76.12		0.74	0.31	1.77	0.501
**Hospital ownership**							< 0.001				
Non-public	4092	69.29	1029	25.15	3063	74.85		–	–	–	–
Public	1814	30.71	493	27.18	1321	72.82		0.83	0.67	1.03	0.089

*MDT* multidisciplinary team, *HR* hazard ratio, *NTD* new Taiwan dollar.

Table 4 presents a relatively low death risk among patients with stages 2, 3, and 4 cancer who adopted the MDT treatment strategy compared with their same-stage counterparts without an MDT. The effect of an MDT was the most substantial among patients with stage 3 cancer, with the death rates for those adopting and not adopting the MDT treatment strategy being 70.95% and 77.75%, respectively. The patients who adopted the MDT treatment strategy exhibited a lower death rate than did those not adopting the MDT treatment strategy, regardless of the service volume of their physician. Regarding the hospital level, among patients visiting medical centres, those with an MDT (68.81%) showed a substantially lower death rate than did those without an MDT (76.38%). Regarding the ownership of the main hospital that each patient visited, an MDT was observed to considerably reduce patients’ death rate in public hospitals (with MDT: 67.49%; without MDT: 77.97%).

**Table 4:** Comparison of the survival of esophageal patients between MDT participants and non-participants.

Variables	Non-MDT						*P* value	MDT						*P* value
	Total		Survival		Death			Total		Survival		Death		
	N	%	n_1_	%	n_2_	%		N	%	n_1_	%	n_2_	%	
**Total**	2953	100.00	698	23.64	2255	76.36		2953	100.00	824	27.90	2129	72.10	< 0.001
**Gender**							< 0.001							< 0.001
Female	178	6.03	65	36.52	113	63.48		195	6.60	80	41.03	115	58.97	
Male	2775	93.97	633	22.81	2142	77.19		2758	93.40	744	26.98	2014	73.02	
**Age at diagnosed (year)**							0.003							0.156
< 45	248	8.40	54	21.77	194	78.23		238	8.06	54	22.69	184	77.31	
45–54	990	33.53	201	20.30	789	79.70		976	33.05	263	26.95	713	73.05	
55–64	1026	34.74	280	27.29	746	72.71		1020	34.54	304	29.80	716	70.20	
65–74	418	14.16	99	23.68	319	76.32		447	15.14	134	29.98	313	70.02	
≧ 75	271	9.18	64	23.62	207	76.38		272	9.21	69	25.37	203	74.63	
**Monthly salary (NTD)**							< 0.001							< 0.001
≦ 17,280	834	28.24	148	17.75	686	82.25		772	26.14	168	21.76	604	78.24	
17,281–2,2800	1202	40.70	299	24.88	903	75.12		1219	41.28	326	26.74	893	73.26	
22,801–28,800	231	7.82	63	27.27	168	72.73		241	8.16	84	34.85	157	65.15	
28,801–36,300	261	8.84	65	24.90	196	75.10		249	8.43	75	30.12	174	69.88	
36,301–45,800	283	9.58	89	31.45	194	68.55		315	10.67	107	33.97	208	66.03	
≧ 45,801	142	4.81	34	23.94	108	76.06		157	5.32	64	40.76	93	59.24	
**Charlson comorbidity index**							0.046							0.559
0	1835	62.14	421	22.94	1414	77.06		1775	60.11	496	27.94	1279	72.06	
1	683	23.13	175	25.62	508	74.38		685	23.20	188	27.45	497	72.55	
2	245	8.30	57	23.27	188	76.73		280	9.48	77	27.50	203	72.50	
≧ 3	190	6.43	45	23.68	145	76.32		213	7.21	63	29.58	150	70.42	
**Cancer stage**							< 0.001							< 0.001
I	116	3.93	71	61.21	45	38.79		120	4.06	73	60.83	47	39.17	
II	558	18.90	212	37.99	346	62.01		596	20.18	233	39.09	363	60.91	
III	1618	54.79	360	22.25	1258	77.75		1597	54.08	464	29.05	1133	70.95	
IV	661	22.38	55	8.32	606	91.68		640	21.67	54	8.44	586	91.56	
**Treatment**							< 0.001							< 0.001
Surgery	312	10.57	138	44.23	174	55.77		164	5.55	80	48.78	84	51.22	
Radiotherapy	172	5.82	19	11.05	153	88.95		131	4.44	14	10.69	117	89.31	
Chemotherapy	149	5.05	28	18.79	121	81.21		98	3.32	16	16.33	82	83.67	
Surgery + radiotherapy	140	4.74	24	17.14	116	82.86		147	4.98	35	23.81	112	76.19	
Surgery + chemotherapy	166	5.62	31	18.67	135	81.33		117	3.96	27	23.08	90	76.92	
Radiotherapy + chemotherapy	877	29.70	171	19.50	706	80.50		772	26.14	204	26.42	568	73.58	
Surgery + radiotherapy + Chemotherapy	1137	38.50	287	25.24	850	74.76		1524	51.61	448	29.40	1076	70.60	
**Service volume of physicians**							< 0.001							< 0.001
Low (< 25%)	798	27.02	132	16.54	666	83.46		694	23.50	152	21.90	542	78.10	
Middle (25–75%)	1484	50.25	365	24.60	1119	75.40		1625	55.03	442	27.20	1183	72.80	
High (> 75%)	671	22.72	201	29.96	470	70.04		634	21.47	230	36.28	404	63.72	
**Hospital level**							0.010							< 0.001
Medical centers	1571	53.20	371	23.62	1200	76.38		1584	53.64	494	31.19	1090	68.81	
Non-medical centers	1382	46.80	327	23.66	1055	76.34		1369	46.36	330	24.11	1039	75.89	
**Hospital ownership**							0.148						< 0.001	
Non-public	2025	68.57	493	24.35	1532	75.65		70.00	536	25.93	1531	74.07		
Public	928	31.43	205	22.09	723	77.91		30.00	288	32.51	598	67.49		

MDT participant observation period:1.59 ± 1.39 years (median: 1.14 years); Non-participant observation period:1.34 ± 1.38 years (median: 0.87 years).

*MDT* multidisciplinary team, *NTD* New Taiwan Dollar.

### Other relevant factors affecting the survival of oesophageal cancer patients

A conditional Cox proportional hazard model (Table 3) was performed to observe the relative risk of survival in patients who adopted and did not adopt the MDT treatment strategy as well as to explore relevant factors affecting patient survival. The following factors had a significant effect on patients’ survival (*p* < 0.05): gender, monthly salary, urbanization level, other catastrophic illnesses, oesophageal cancer stage, treatment methods, and service volume of the physician. By contrast, patients’ age, CCI, hospital level, and hospital ownership showed no significant effect on patient survival (*p* > 0.05). In the Table 3, the death risks of all age groups were greater than 70% in the studied period. Those who developed oesophageal cancer at an age less than 45 years had the highest death risk (77.78%), followed by those who developed the cancer at 75 years or older. However, with relevant variables controlled for, the death risk of those who developed cancer at each age group was not significantly different from that of those who developed cancer at less than 45 years (*P* > 0.05). The analysis of patients’ socioeconomic status revealed the highest death rate (80.32%) among those whose monthly salary was NT$17,280 or lower. With relevant variables controlled for, the death risk of patients with a monthly salary of NT$28,801–36,300 was 0.65 times that of patients with a monthly salary of NT$17,280 or lower (95% CI 0.45–0.94). Patients with a monthly salary of NT$45,801 or higher had a death risk 0.42 times that of patients with a monthly salary of NT$17,280 or lower (95% CI 0.23–0.75). Regarding the health status, the death risk of patients with other catastrophic illnesses was 1.78 times that of patients without such an illness (95% CI 1.16–2.74). In the study period between 2010 and 2015, the survival rate of patients with stage 1 oesophageal cancer was 61.02%; the survival rate was significantly lower in patients with stage 2 cancer (38.56%); that of patients with stage 3 cancer was 25.63%; and that of patients with stage 4 cancer was extremely low at 8.38%. With relevant variables controlled for, the death risk increased as cancer progressed: the death risks of patients with stages 3 and 4 cancer were respectively 2.75 (95% CI 1.78–4.26) and 6.49 (95% CI 3.88–10.85) times that of patients with stage 1. According to the analysis of the service volume of the physician, patients whose physician had a higher service volume showed a lower death risk. The death risk of patients whose physician had a high service volume was 0.81 times that of patients whose physician had a low service volume (95% CI 0.67–0.97).

## Discussion

### Characteristics of patients with oesophageal cancer adopting and not adopting the MDT treatment strategy

This study is the first nationwide cohort research discussing the effect of MDTs of the survival results of newly diagnosed oesophageal cancer patients and the characteristics of such patients who adopted and did not adopt the MDT treatment strategy. This study recruited 14,563 patients newly diagnosed with oesophageal cancer from 2010 to 2015, among which 12,908 had received a surgery, chemotherapy, or radiotherapy within 1 year of their diagnosis. Subsequently, patients who adopted the MDT treatment strategy were matched at a ratio of 1:1 with those who did not adopt the MDT treatment strategy, finalising the sample to 2953 patients with MDT care and 2953 without MDT care. According to the results, whether patients adopted the MDT treatment strategy was associated with the following factors (*p* < 0.05): monthly salary, urbanization level of residence, cancer stage, level of the main hospital visited, and service volume of the physician. Studies have verified the association between whether a patient adopted the MDT treatment strategy and the patient’s disease severity, level of the main hospital visited, and the service volume of the physician^5,11^. The proportion of patients with a high monthly salary who adopted the MDT treatment strategy was higher compared with their low-monthly-salary counterparts, which was probably because patients with higher income and their families had a stronger will and better ability to seek medical help. The proportion of patients living in high-urbanization-level areas who adopted the MDT treatment strategy was higher than patients living in low-urbanization-level areas; this may be because hospitals with a sufficient scale and capacity to practice MDTs were mostly located in high urbanization level regions.

### Effect of MDTs on the survival of oesophageal cancer patients

The proportion of people with cancer has been growing over the last few years. Globally, much research attention has been paid to oesophageal cancer in particular—the world’s sixth most common cause of cancer death. Prior researches had predominantly examined risk factors of oesophageal cancer or the effect of different treatments on patient survival^24–26^. By the time of writing the present study, only one small-scale before–after study focused on the effect of MDT intervention on the survival of oesophageal cancer patients^10^. Since there is a lack of strong evidence to support the effectiveness of MDTs for oesophageal cancer patients, investigating data from the TCRD and NHIRD, this nationwide, retrospective cohort research was performed to analyse the effect of MDT intervention in improving survival rate of patients with oesophageal cancer.

An MDT is aimed at benefiting both medical service providers and patients, improving the satisfaction and psychological state of patients and bringing together relevant medical providers for the joint formulation of care plans. These features have contributed to the growing popularity of MDTs in countries such as the United Kingdom, the United States, Australia, and European countries^9^. By incorporation of MDTs into the care plan for cancer patients improves the results of medical care. This is mostly because MDT intervention is a shift from the conventional care model that involves only a single medical department towards an integrative care model that engages specialists from relevant departments, including physicians, surgeons, oncologists, pathologists, radiologists, dietitians, physiatrists, nurses, and social workers, and MDT intervention involves regular meetings among these specialists to discuss and follow-up the status of patients with cancer^8^. In particular, due to the high complexity involved and the high probability of comorbidities, the diagnosis and treatment processes for cancer patients require a combination of various diagnostic and treatment methods. Accordingly, MDTs facilitate collaboration among medical teams, enhance compliance with treatment guidelines, reduce inappropriately treatment recommendations, shorten diagnostic time, boost the accuracy of diagnoses, and thus increase the survival of patients with cancer^5–7,22^.

In this study, we used the 1:1 propensity score matching method to minimize the selection bias between the two groups of patients (with and without an MDT) for eliminating the effects of confounding factors on the patients’ adoption of the cancer MDT strategy. As shown in Table 3, the death risk of patients adopting the MDT treatment strategy was 0.73 times that of patients not adopting the MDT treatment strategy. Accordingly, with relevant factors controlled for, patients with oesophageal cancer adopting the MDT treatment strategy had a lower death risk than did those not adopting the MDT treatment strategy. The study results are consistent with the finding of various studies that MDTs improved the survival rate in patients with different cancers^5,11,17,27^. However, only one study discussed the effect of MDT intervention on the survival results of oesophageal cancer patients; that research was performed in a medical institution in the United Kingdom from 1991 to 2003 and had a study population of only 144 patients. The same study revealed that MDT intervention improved the survival results of oesophageal cancer patients, with an increase of the 5-year survival rate from 10 to 52% (*p* < 0.05). Nevertheless, the before-after design of the study created various research limitations and hindered the generalisability of the results^10^. Studies in oral, gastric, lung, colorectal, breast and ovarian cancers have all indicated a beneficial effect of MDTs on clinical care outcomes^5,14,27–29^.

### Associated factors of the survival of oesophageal cancer patients

According to Table 3, the survival of oesophageal cancer patients was significantly affected (*p* < 0.05) by the demographic factor (gender), health status (other catastrophic illnesses), socioeconomic factor (monthly salary), environmental factor (urbanization level), cancer stage, treatment, and service volume of the physician.

The study results showed that male patients had a death risk 1.45 times higher than female patients (95% CI 1.01–2.09), which is consistent with the findings of a previous study^30^. Patients with a higher monthly salary exhibited a lower death risk; specifically, the death risk of those with a monthly salary of NT$45,801 or more was 0.42 times that of those with a monthly salary of NT$17,280 or less (95% CI 0.23–0.75). Hence, socioeconomic factors affect the survival of oesophageal cancer patients. The National Programme of Cancer Registries Patterns of Care Study lead by American scholar Byers argued that a low socioeconomic status results in a less favourable prognosis in patients with cancer; this is because a low socioeconomic status may lead to delayed diagnoses and passive cancer treatment^31^. In Taiwan, cancer is categorised as a catastrophic illness as per the National Health Insurance Act; thus, patients with cancer are partially exempt from covering the medical costs involved in treating cancers. Despite the exemption, the association between a low socioeconomic status and unfavourable prognosis in patients with cancer remained in the present study. Another Taiwanese study also revealed better prognosis in high-socioeconomic-status patients with oesophageal cancer than in their low-socioeconomic-status counterparts^32^. Additionally, high-socioeconomic-status patients with oesophageal cancer, even if living in a low-socioeconomic-status area, had a higher chance of receiving esophagectomy^32^.

According to the analysis of patients’ health status, those with other catastrophic illnesses had a death risk that was 1.78 times higher than that of those without such illnesses (95% CI 1.16–2.74). This finding is consistent with previous findings^33^. Regarding the cancer stage, patients at a later cancer stage had a higher death risk; the death risks of patients with stage 3 and stage 4 cancer were respectively 2.75 (95% CI 1.78–4.26) and 6.49 (95% CI 3.88–10.85) times that of patients with stage 1 cancer. The prognosis of oesophageal cancer had a close association with the cancer stage of a patient at diagnosis; those in the early stage at diagnosis exhibited more favourable prognoses^34^. In Taiwan, oesophageal cancer is usually diagnosed at stage 2, 3, or even 4; thus, the prognosis is mostly unfavourable. Despite advancements in surgical techniques and postoperative care over recent years, the postoperative 5-year survival rate for oesophageal cancer has improved only slightly by 10–20%^35^.

According to analysis results for the service volume of the physician, a low death risk was observed among patients whose physicians had a high service volume; specifically, these patients had a death risk 0.81 times that of patients whose physicians had a low service volume (95% CI 0.67–0.97). Studies have demonstrated a positive effect of physicians’ service volume on patient care outcomes, which is probably attributable to the abundant care experience accumulated and excellent techniques honed by providing services and performing a large number of surgeries. Research has also shown that for physicians or hospitals with a high service volume, medical teams with abundant experience possess a higher skill level and better ability to execute treatment plans, in turn contributing to more favourable treatment outcomes and a lower patient death risk^36,37^. Previous research and the present study propose the consistent finding that high service and surgery volumes of physicians are associated with a low death rate of patients.

There were limitations in this study. First, our research collected data from the TCRD and NHIRD, and the discussion was limited to variables contained in the two databases. Therefore, other factors (e.g., smoking, drinking, or exercise habit) could not be included in this study, which are potentially related to the survival results of oesophageal cancer patients. Second, our research did not classify patients into groups with different pathological cell types, which could have contributed to the different survival rates. Third, the databases did not reveal whether patients had completed the entire treatment.

In conclusion, MDT intervention significantly reduced the death risk of patients with oesophageal cancer (HR = 0.73). Patients with the following characteristics had a less favourable prognosis: males, low socioeconomic status, presence of other catastrophic illnesses, low service volume of the physician and late stage of cancer.

## Materials and methods

### Data source

In this retrospective nationwide cohort study, we investigate data of oesophageal cancer patients from the TCRD, and mortality outcome from Cause of Death Data which were released by the Ministry of the Interior. Then, the NHIRD data from 2008 to 2016 were used for subsequent analysis of relevant variables. NHIRD contains comprehensive healthcare data of more than 23 million civilians who were representative of 99.7% of the residents of Taiwan^11^.

### Study design

We explored the TCRD during January 2010 to December 2015 for oesophageal cancer patients and the Cause of Death Data during January 2010 to December 2016 for mortality outcome. The study cohort retrieved patients with incidental oesophageal cancer (ICD-9-CM: C150) who received treatment in a hospital for their oesophageal cancer within a year after oesophageal cancer was diagnosed. The treatments included surgical treatment, chemotherapy or radiation. The exclusion criteria include.

1. Patients who had other coexisting cancers or who had developed other cancers were excluded: Because this study examined survival rate differences between patients with oesophageal cancer who adopted and did not adopt the MDT treatment strategy, the presence of other cancers could exert an influence on the survival of these patients.
2. Patients receiving palliative were excluded: This study explored survival rate differences between patients with oesophageal cancer who adopted and did not adopt the MDT treatment strategy. Therefore, including patients who received palliative treatment after the diagnosis, which indicates the absence of curative intent treatment, in the comparison between the two aforementioned groups is inappropriate.
3. End-stage patients with mortality outcome within a month after the diagnosis were excluded: These patients may not be able to receive MDT treatment in time to reflect the benefits of MDTs.
4. Patients with stage 0 oesophageal cancer were excluded: According to an American study, the 5-year survival rate of stage 0 oesophageal cancer patients is higher than 90%^38^. Additionally, stage 0 oesophageal cancer is usually diagnosed and treated by specialists of a single medical department. Therefore, the intervention of MDTs may not provide much benefit to the survival results of stage 0 oesophageal cancer patients, who were thus excluded from the study.

**Figure 3:**
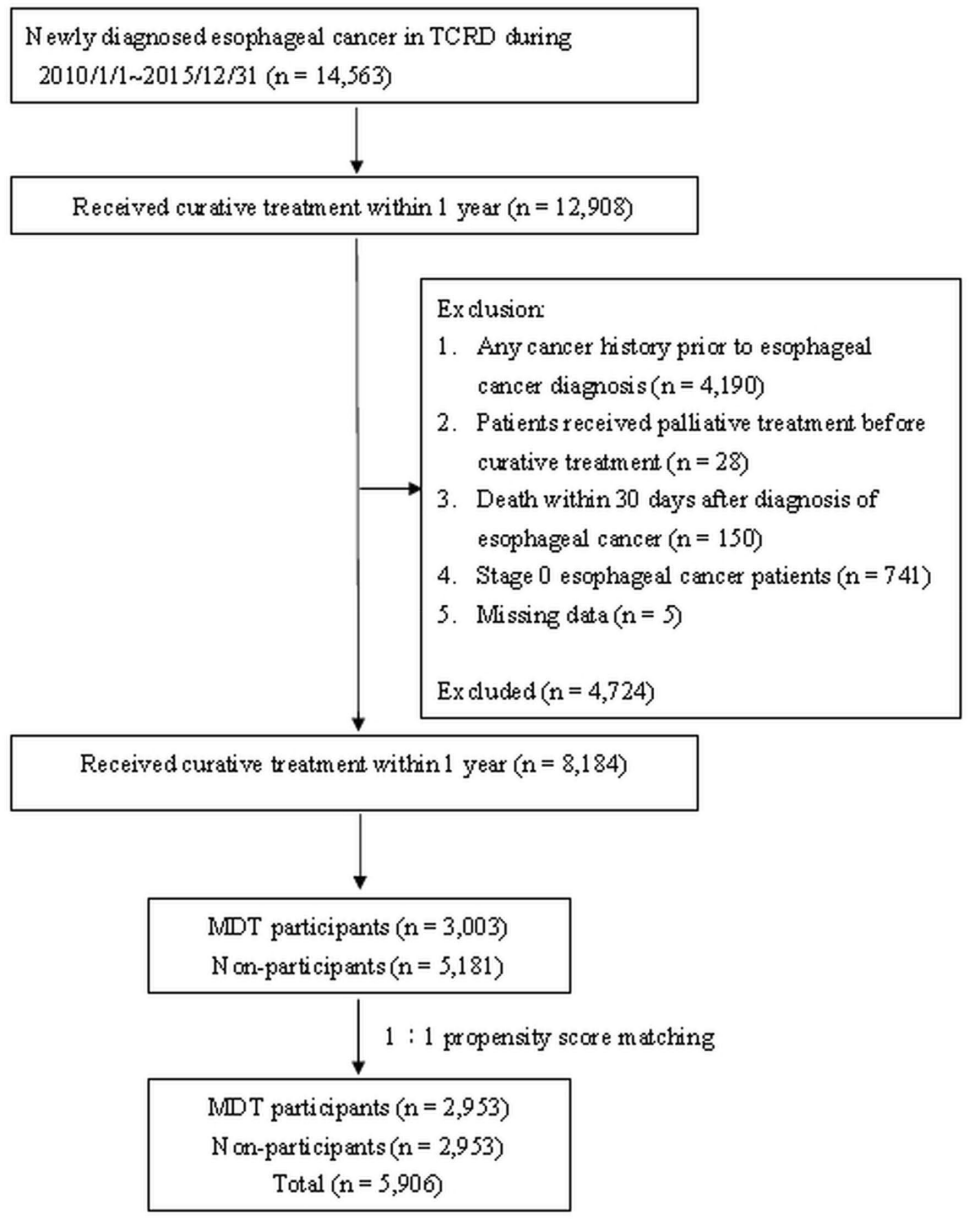
Flowchart of MDT participants and non-MDT participants enrolled from Taiwan Cancer Registry Database in Taiwan during 2010–2015.

To mitigate the selection bias between the groups adopting and not adopting the MDT treatment strategy, this study employed 1:1 propensity score matching and a logistic regression model to predict whether a patient would adopt the MDT treatment strategy or not. The matching approach was used to control for the effects of confounding factors on the adoption of MDTs in the treatment plan, thereby increasing the consistency between patients adopting and not adopting the MDT treatment strategy. The selection process of study participants is showed as in Fig. 3.

Since the patient identifications in the National Health Insurance Research Database have been scrambled and de-identified by the Taiwan government for academic research use, the informed consent was waived by the Research Ethics Committee of the Changhua Christian Hospital. The research was conducted in accordance with the 1964 Declaration of Helsinki and amendments and was approved by the institutional review board of the Changhua Christian Hospital (IRB No. 181259), Taiwan.

### Variables of interest

Since medical providers in Taiwan can have revenues by MDT care with appropriate medical records, this study claimed the aforementioned information to defined the MDT group. The control variables were as follows: demographic characteristics (age and sex), socioeconomic factor (monthly salary), environmental factor (urbanization level), health status (comorbidities, cancer stage and other catastrophic illnesses), and the features of the main hospital and physician visited (hospital ownership, hospital level, and service volume of the physician).

The urbanization level of patients’ workplace and residence was determined according to the locations of units from where health insurance was purchased for them and with reference to ‘Incorporating Development Stratification of Taiwan Townships into Sampling Design of Large Scale Health Interview Survey’ by Liu et al. in 2006. Liu et al. classified urbanization into Levels 1 to 7 by conducting a cluster analysis with the following variables: proportion of the population with a junior college degree or higher, population density, proportion of the population 65 years or older, proportion of the population working in agriculture, and number of Western medicine doctors per 100,000 residents. Level 7 represents the least urbanized areas; otherwise, level 1 represents the most urbanized areas^39^.

Regarding the health status, the comorbidities of patients were classified into 17 categories in accordance with Deyo’s CCI. ICD-9-CM codes commissioned to the principal and additional diagnoses of patients were converted into weighted scores, which were then summed to obtain the CCI score^40^. CCI scores were classified into the following four levels in this study: 0, 1, 2, and 3 or higher.

The cancer stage referred to the stage of cancer at the time of diagnosis defined by the American Joint Committee on Cancer, which comprised four stages, namely stages 1, 2, 3, and 4.

The main hospitals visited referred to the medical institutions where each of the patients was diagnosed with oesophageal cancer. The hospitals were classified into three hospital levels (i.e., medical centres, regional hospitals, and district hospitals) and two ownership types (i.e., public and private hospitals).

Definition of the service volume of the physician refers to the numbers of oesophageal cancer patients treated by the physician of each studied patient in the year when the patient received treatment for oesophageal cancer. For subsequent analyses, the service volume was divided by quartiles into the following three levels: low (lower than 25%), medium (25–75%), and high (higher than 75%).

### Main outcome measurements

The dependent variable, namely whether patients with oesophageal cancer survived or not, was determined by obtaining the dates of death of patients from the Cause of Death Data from January 2010 to December 2016. With relevant variables controlled for, adjusted survival curves for patients with and without an MDT were generated.

### Statistical analyses

This retrospective and longitudinal cohort study used SAS 9.4 for data organisation and statistical analyses. A Chi-square test was performed to determine whether oesophageal patients adopting or not adopting MDT care were statistically different in terms of the following variables: demographic characteristics (age and sex), socioeconomic factor (monthly salary), environmental factor (urbanization level), health status (comorbidities and other catastrophic illnesses), cancer stage, treatment methods, and main hospital visited (hospital level and hospital ownership).

To minimize the selection bias between study subjects adopting and not adopting the MDT treatment strategy, propensity score matching was conducted at a 1:1 ratio. Logistic regression was executed to build a model. The dependent variable was whether patients adopted the MDT treatment strategy, and the independent variables were the demographic characteristics (age and sex), socioeconomic factor (monthly salary), environmental factor (urbanization level), health status (comorbidities and other catastrophic illnesses), cancer stage, treatment methods, and main hospital visited (hospital level and hospital ownership). Accordingly, propensity score matching method was performed to control for the effects of confounding factors on patients’ adoption of the MDT treatment strategy and thereby enhanced the consistency between patients adopting and not adopting the MDT treatment strategy. A Chi-square test was then performed to examine differences of different variables between those adopting and not adopting the MDT treatment strategy.

A conditional Cox proportional hazard model, with relevant variables controlled for, was conducted to determine the relative risk of survival of patients adopting and not adopting the MDT treatment strategy on a weekly basis. The analysis result was presented in HR and 95% CI. The dependant variable was whether a patient survived or not; the independent variable was whether a patient adopted the MDT treatment strategy; and the control variables were a patient’s demographic characteristics, socioeconomic factor, environmental factor, health status, and oesophageal cancer stage, treatment methods, as well as the features of the main hospital and physician visited. With relevant variables controlled for, adjusted survival curves were generated to present differences between the survival curves of patients who adopted and did not adopt the MDT treatment strategy.

## Data Availability

Regarding the data availability, data were obtained from the National Health Insurance Research Database published by the Ministry of Health and Welfare, Taiwan. Due to legal restrictions imposed by the Taiwan government related to the Personal Information Protection Act, the database cannot be made publicly available. All researchers can apply for using the databases to conduct their studies. Requests for data can be sent as a formal proposal to the Health and Welfare Data Science Center of the Ministry of Health and Welfare (http://www.mohw.gov.tw/EN/Ministry/Index.aspx). Any raw data are not allowed to be brought out from the Health and Welfare Data Science Center. The restrictions prohibited the authors from making the minimal data set publicly available.
